# Collaboration: The Force That Makes the Impossible Possible

**DOI:** 10.1016/j.adro.2022.100966

**Published:** 2022-05-10

**Authors:** Manjit Dosanjh

**Affiliations:** a CERN, Geneva, Switzerland; b Department of Physics, University of Oxford, Oxford, United Kingdom

## Abstract

Over the last three decades, the landscape of cancer treatment with radiotherapy has never stopped improving. ENLIGHT – the European Network for Light Ion Hadron Therapy – has been an active participant in the huge changes that have taken place, in particular in Europe. At the end of the 90s when I arrived at CERN, it appeared clear that an improvement in communication, sharing and exchange, while keeping a common goal, was needed to bring together international experts from accelerator physics, imaging, medical physics, radiobiology and clinical medicine. ENLIGHT network was most aptly launched at CERN, since CERN is renowned as a place for global collaboration. The network has come a long way since the kick-off meeting at CERN in 2002 when only about 70 specialists from different disciplines took part and continues to grow and flourish with now over 1000 participants, accounting for over 100 institutions, from around 40 countries around the globe.

For the past 3 decades, the landscape of cancer treatment with radiation therapy has never stopped improving. The European Network for Light Ion Hadron Therapy (ENLIGHT) has been an active participant in the huge changes that have taken place, particularly in Europe.

We were not first in feeling the need for networking. Indeed, since the 1980s, physicians and scientists coming from a variety of fields (medical physicists but also radiobiologists, engineers, etc) were actively exchanging information and best practices when they were dealing with nonconventional radiation therapy techniques. At the beginning, those exchanges and interactions focussed on the neutron radiation therapy more than on proton therapy, which was not yet taking off. One of the first networks in Europe was the European Organisation for Research and Treatment of Cancer Heavy Particle Therapy Group, which became later the European Heavy Particle (Hadron) Therapy Group.

Later on, in the 1990s, the focus started to shift toward the development of initiatives in proton and carbon ion radiation therapy. Those initiatives were taking place in the laboratories for accelerator and particle physics, including, of course, the European Organization for Nuclear Research (CERN), where in 2000, I was asked to lead the effort aiming at promoting the pioneering Proton-Ion Medical Machine Study (PIMMS)^1^ study as standard for hadron therapy centers (Fig 1). ENLIGHT network was most aptly launched at CERN since CERN is renowned for global collaboration.^1^

**Figure 1:**
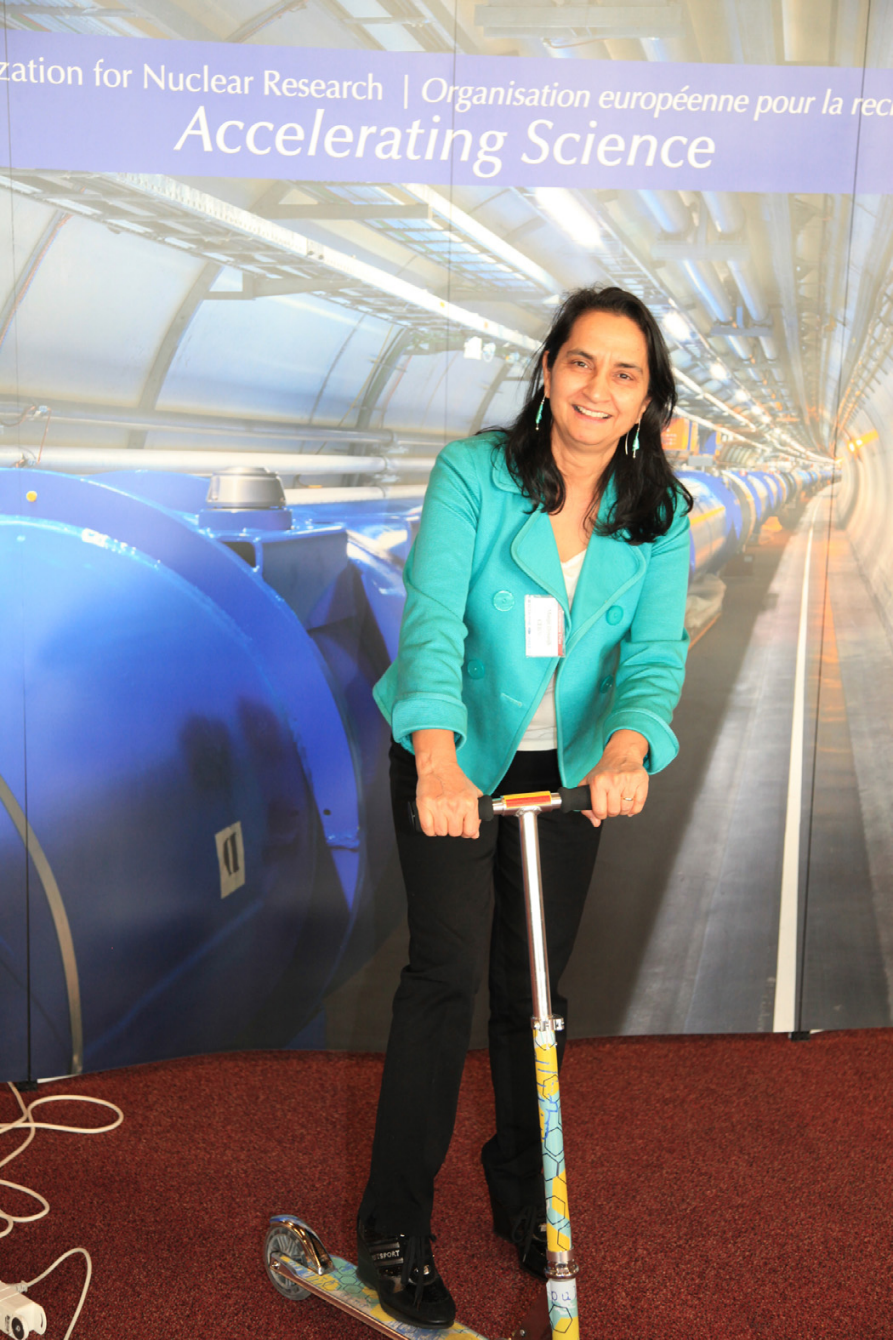
Moving toward the next 20 years of the European Network for Light Ion Hadron Therapy because “life is like riding a bicycle, to keep your balance, you must keep moving.” Albert Einstein shared these words of advice with his son in a letter dated February 5, 1930.

## Landing at CERN

In 2000, nearly 50 years after its foundation, CERN was the leading scientific laboratory in the world and not just for particle physics. The laboratory was home to a huge number of innovative activities, and it was in this framework that I was asked to coordinate the various efforts and build bridges between physics and life sciences and medical communities.

At CERN, people are used to collaborating, actually collaboration has become second nature for particle physicists, it is almost part of their DNA. They find it normal to establish multinational teams reach consensus to achieve ambitious goals, which cannot be achieved individually. When I arrived, there were more than 4500 users collaborating from nearly 260 institutes from European countries and more than 1600 from around 200 institutes around the world. It was also the time when the laboratory was switching off the Large Electron-Positron accelerator and preparing for the Large Hadron Collider, which would later enable the Nobel prize winning search for the Higgs boson.

In addition, after many years of research and development in accelerators mostly for physics, CERN had coordinated the PIMMS study which had just come to completion, ready for translation and exploitation into the medical arena. It was at this exciting transition period I came to be the catalyst whose goal was to connect all the dots.

## Exotic Species

In 2000, CERN had just established a technology transfer unit and the newly appointed director had the ambition to show the relevance of particle physics to the society at large with a specific focus on the medical community. As a first and unique biologist (often called “exotic species” by Management) hired specifically to facilitate the implementation of CERN applications, my task was to identify the relevant technologies and build bridges for translating these into the medical field and have impact on society.

It quickly became clear that detectors such as Medical Pixel detector, Compton Camera, Crystals, Gas Electron Multipliers, Imaging Silicon Pixel Array and accelerators such as Linac-Booster and PIMMS could have a direct effect, but the question was how to make it happen.

Ugo Amaldi, Ettore Rosso, Michal Campbell, Erik Heine, Paul Lecoq, Fabio Sauli, and Peter Weihammer were the CERN scientists involved in key technologies for which I could see a direct application in the medical field. They wanted to try and translate them for hospital use, but they did not have the necessary knowledge of how the medical community works or what its needs were. For example, the detection devices that CERN had were powerful technologies that performed in terms of nanometers (ie, high precision) and nanoseconds (ie, fast process), whereas doctors were familiar with equipment that worked with millimeters and milliseconds.

In the high-energy physics field, we are always striving for the highest performance, the cutting-edge machines. These tools are, in general, unique machines, which inevitably need complex operation and maintenance; they are run by physicists or engineers, and they are not for commercial production (although industry acts as manufacturer). In contrast, in the biomedical environment, the need for robustness, ease of operation, and minimal maintenance is a must, and industry is a partner for both production and distribution.

From the beginning, I was very much aware of that underlying deep gap existing between the 2 communities as well as the subsequent challenges that were lying ahead of me. Of primary importance was the creation of a collaborative environment where the two communities could brainstorm, build trust, exploit knowledge, and fulfil the need. The story starts with the language.

## Mind the Gap

Hadron therapy: the word itself means a lot for physicists but nothing to the medical community. The medical community wants to use and is using “particle therapy.” On the other hand, physicists want to highlight the differences among the different particles (electrons, photons) and they want to highlight the advantages that hadron therapy can bring to cancer treatment.

**Figure 2:**
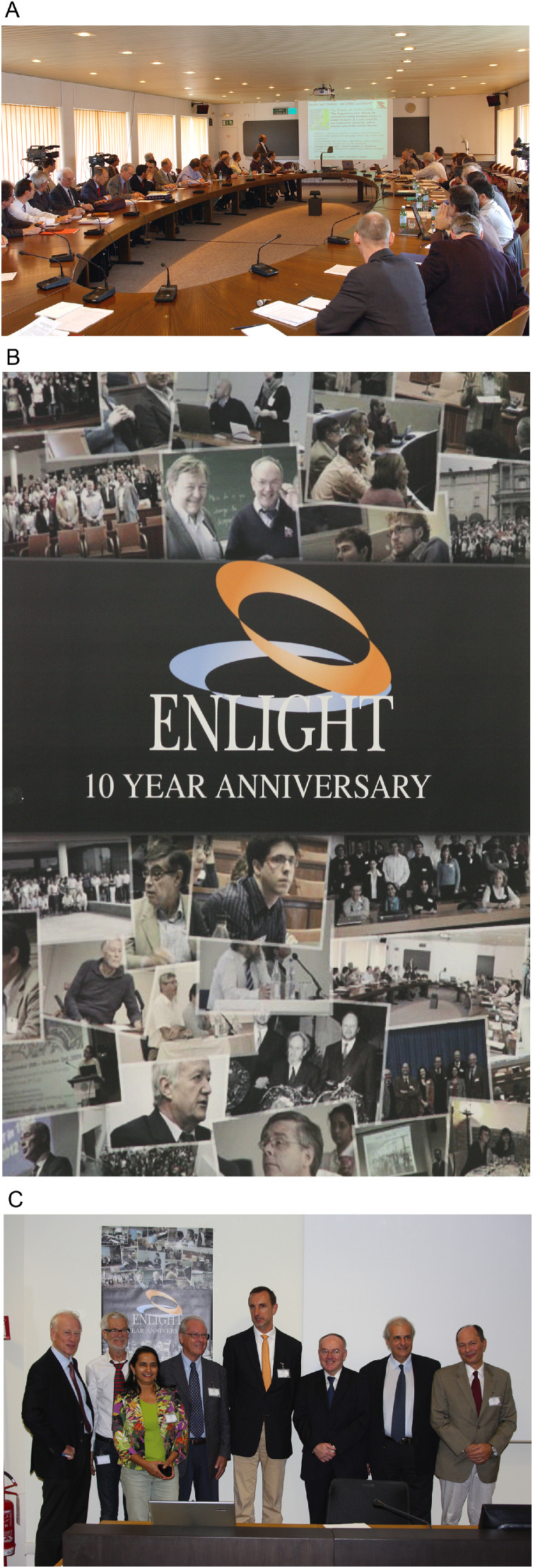
A, The participants from all interested European institutes, research centers and clinics lead by Jean Pierre Gérard, the then president of ESTRO, at the launch of the the European Network for Light Ion Hadron Therapy network. The event was supported by the European Organization for Nuclear Research and the then Director for Technology Transfer, Hans Hoffman, was key for our initiative. Ugo Amaldi (of TERA), Jean-Pierre Gérard and Germaine Heeren (of ESTRO), Gerard Kraft (of GSI), Hans Svensson and Anders Brahme (of KI), Richard Pötter (of MedAustron), Jürgen Debus (of Heidelberg), Wolfgang Enghardt (of Dresden), Luciano Maiani (of the European Organization for Nuclear Research DG) were some of the names that presented and envisioned the dynamic collaboration that continues to work today. Gerard's phrase of 3Cs (Cheap, Conservative, Cure) has been often requoted since that day. B and C, In 2012, the European Network for Light Ion Hadron Therapy celebrated its 10-year anniversary, and it seemed like a dream. Ten years later, we can say that building a multidisciplinary community of youngsters, experts, and leaders in the field of hadron therapy and fostering collaborations all over Europe and worldwide is even more empowering. *Abbreviations:* DG = director general; ESTRO = European Society Radiation Oncology; GSI = GSI Helmholtzzentrum Fuer Schwerionenforshung; KI = Karolinkska Institute; TERA = Fondazione per Adroterapia Oncologica.

The use of heavy and light particles used for radiation therapy also emphasizes another cultural difference: carbon is heavy for biologists and clinicians but light for physicists to the point that in the ENLIGHT kick-off meeting, during the press release, the physics community complained because the description of the German Heavy Ion Project. They were wondering, “when did carbon become heavy?” The resulting clinical facility is called the Heidelberg Ion Therapy Center (HIT). Along the same lines, the name ENLIGHT (containing “light ion hadron therapy”) is another case where the biologists are wondering, “why light?” (Fig 2A).

Learning to overcome these differences to bridge the culture gap is what makes the field challenging, interesting, stimulating, and attractive. The goal for everybody is to understand the translational need “from laboratory to bed,” as quickly as possible. Over the years, we have moved from x-rays, to particles/hadrons, to very high energy electrons and flash, but, more importantly, we have also leveraged them for peace, diplomacy, and sustainable innovation to benefit the 90% that do not have any access to radiation therapy in developing countries.

If we look only at the physics principles, hadron therapy is undoubtedly more effective (and less harmful) than conventional radiation therapy done with photons; however, building the huge machines and studying the detailed effect on living tissues is a long process that involves huge investments. Did we ever consider those challenges to be unsurmountable? No, because we believed in the 3 pillars: multidisciplinarity, collaboration, and education/training.

Initially, most of the knowledge we could have access to in Europe was coming from Japan for carbon ion radiation therapy facilities and from the United States for proton radiation therapy facilities (especially Berkeley, which is also where I came from before starting my job at CERN).

In the 1990s, the GSI physics laboratory became the first place in Europe to treat patients with carbon ions, resulting in HIT, the first dedicated dual ion hospital-based clinical facility in Europe. It is in this varied landscape that we started to explore uncharted territories in terms of interdisciplinary collaboration rather than trying to reach separately the standards that other countries had reached before us. In 1996, under the CERN umbrella, the PIMMS study was started by the CERN-TERA-MedAustron-Onkologie-2000 collaboration. The study led to the construction of 2 clinical centers: the National Italian Center for Oncologic Hadron Therapy (CNAO) in Pavia, Italy, which treated its first patient in 2011, and MedAustron in Wiener Neustadt, Austria, which treated its first patient in 2016. Although the research centers were setting the standards, private companies also started to enter the game. Today, they certainly play an important role in the diffusion of proton therapy in Europe.

At the end of the 1990s, it appeared clear that an improvement in communication, sharing, and exchange, was needed to bring together international experts with common goals from accelerator physics, medical physics, radiobiology, and clinical medicine. What better place than CERN, whose roots are engraved in international collaboration since its inception? CERN was established in the aftermath of World War II to bring nations and people together for the peaceful pursuit of science. Scientists from all over the world work together with the common goal of advancing science, and collaboration is key to their success.

## The Critical Mass

ENLIGHT saw the light with the signature of a memorandum of understanding in 2001 and the inaugural meeting at CERN in February 2002. We were coordinating the efforts of an initially small group of leading experts: Jean-Pierre Gerard, Ugo Amaldi, Gerhard Kraft, Richard Poetter, Hans Svensson Anders Brahme, and Juergen Debus. From the beginning, our vision was to involve an as varied as possible spectrum of competencies and approaches from across Europe.

The first years of the new network were spent building the critical mass in the consortium and community spirit, and the number of participants continued to grow even after ENLIGHT stopped receiving dedicated networking funds from the European Commission in 2006. Other grants received from the European Union between 2008 and 2015 (a total of 24.6 million Euros) allowed the network to carry out projects that have shaped the European landscape in light ions radiation therapy.^2^, ^3^, ^4^, ^5^ Four projects were submitted under the umbrella of ENLIGHT: Particle Training Network for European Radiation therapy (PARTNER), European Novel Imaging Systems for Ion Therapy (ENVISION), Research Training in 3-Dimensional Imaging for Cancer Radiation Therapy (ENTERVISION) coordinated by me, and the Union of Light Ion Centers in Europe (ULICE) coordinated by Roberto Orecchia of CNAO (Fig 2B and C).

**Figure 3:**
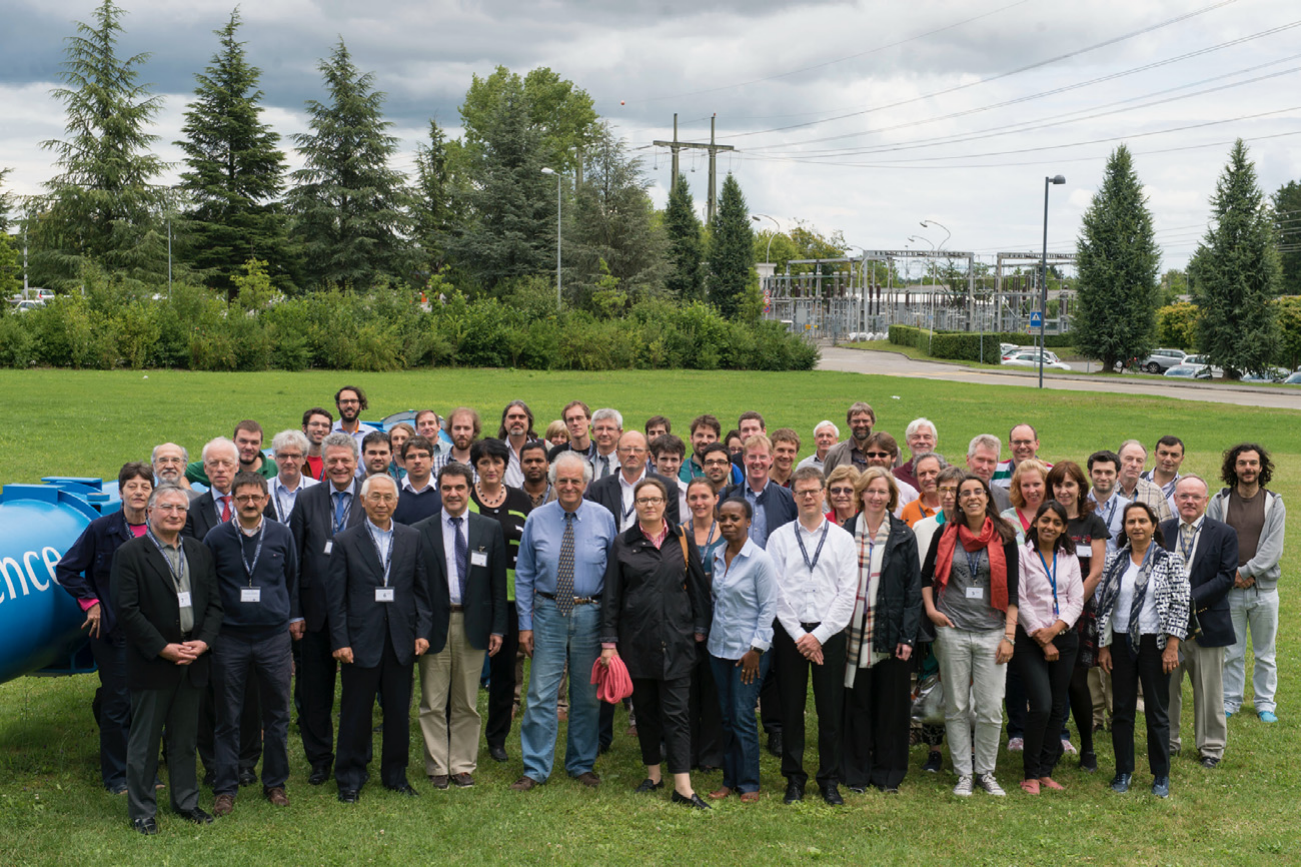
The 2014 annual European Network for Light Ion Hadron Therapy and European Union–funded Union of Light Ions Centers in Europe project meeting was held at the European Organization for Nuclear Research. A special seminar by Dr Eleanor Blakely from the Lawrence Berkley Laboratory celebrated the 60 years of the European Organization for Nuclear Research and the 60 years of when the very first patient was treated at Berkeley. The presence of “Ellie” from Berkeley was especially memorable for me as it brought together my past and present. Twelve years after the launch of the European Network for Light Ion Hadron Therapy network, Union of Light Ions Centers in Europe brought together leading European clinical facilities in hadron therapy.

Today, ENLIGHT has over 1000 participants, accounting for over 100 institutions, from around 40 countries.^7^ The network has come a long way since the kick-off meeting at CERN in 2002, when only about 70 specialists from different disciplines took part. However, that meeting was the seed from which the essence of multidisciplinary collaboration sprang at a time when the various communities—public and private institutions, physicians and physicists, biologists, radiobiologists, and oncologists—were more likely to look at themselves as competitors rather than as collaborators. The change had started and, indeed, is still taking place (Fig 3).

## Conquering Europe

It did not take long to the initial results of our efforts in bringing together the various communities to become visible at a wider scale. Over just one decade, hadron therapy gained a huge amount of momentum in Europe. At the end of 2000, there were only a handful of centers in Europe; today there are 28 of which 4—CNAO, HIT, Marburg, MedAustron—are dual centers. Hadron therapy has grown also across the planet: in 2020, we have 104 centers in operation, and soon there will be 140 centers operating in nearly 30 countries. That means that millions of people across Europe have access to the radiation therapy clinical option to treat cancer. Looking in detail at the number of patients, we can appreciate the effect that such growth had on the overall strategy for treatment of cancer in Europe. As of now, 250,000 patients have been treated globally with protons and 40,000 with carbon, and the demand is still increasing. The approximately 40 new centers currently under construction will help absorb such a demand.

During the past 2 decades, we have raised awareness on how important and key it is to set up a new vision based on collaboration, particularly in fields like radiation therapy in cancer treatment, which is, by its nature, multidisciplinary. But the third pillar, training, was steadily growing in importance since the beginning of ENLIGHT, and again, we borrowed from CERN's foundations.

## Building the Future with Education

During the infancy years of hadron therapy in Europe, one issue appeared very clear: innovative health methods require 360° experts whose training is far from being a straightforward task. Basically, we can say that those specialized experts must result from a mix of physics, engineering, computing, clinical medicine, radiobiology, and, obviously, oncology. And no individual university does this job.

In 2008, ENLIGHT, in the framework of the Seventh Framework Program call, started the 4-year Marie Curie Initial Training Network PARTNER project. The project's goal was to train researchers in the diverse fields that are required to ensure the most effectiveness and efficiency in the patients’ treatment with light ion beams. PARTNER involved 10 institutes and research centers and 2 industrial partners.

**Figure 4:**
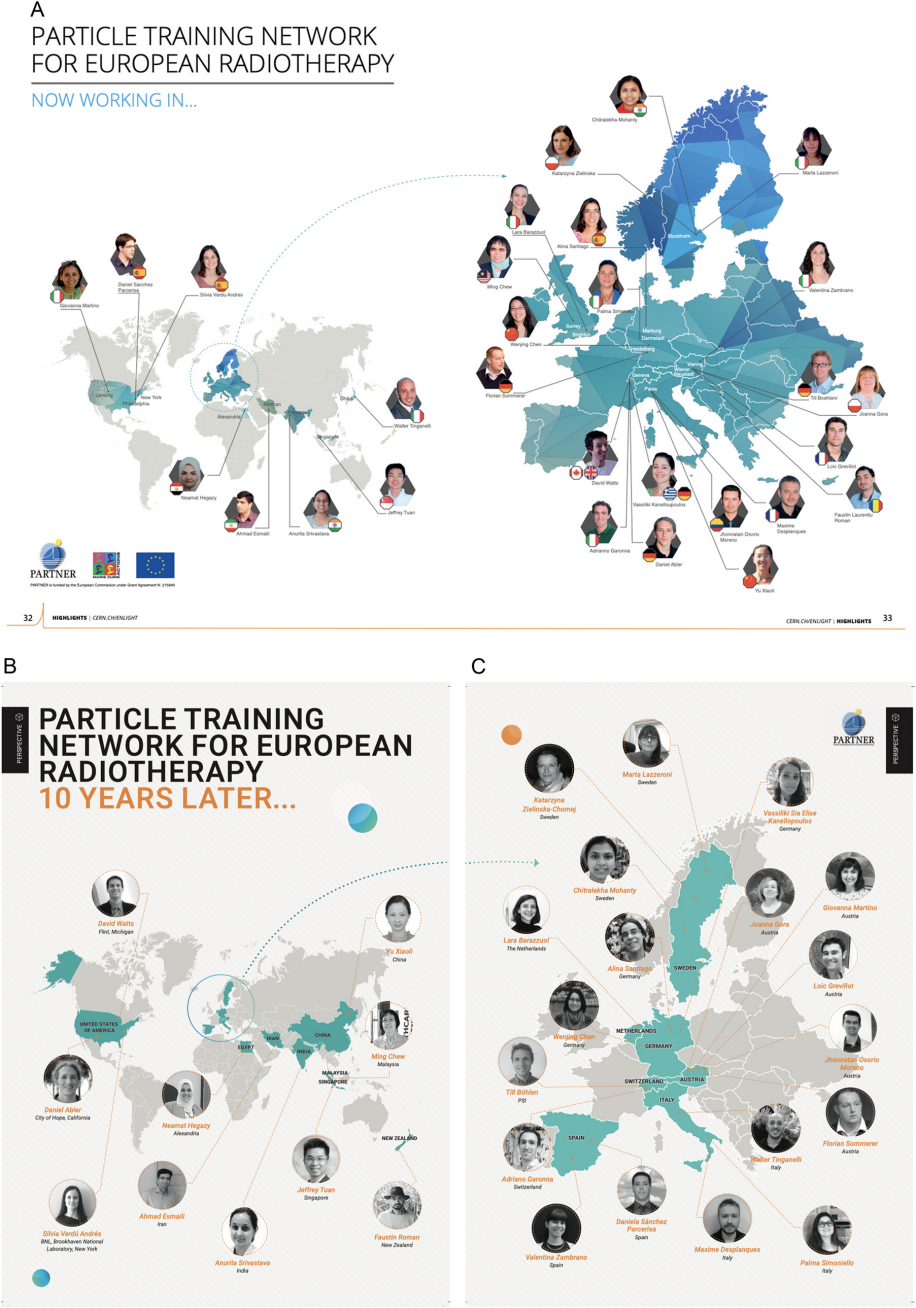
A, In 2008, our Marie-Curie submitted project Particle Training Network for European Radiation therapy was funded, and it was given one of the highest scores and the largest amount of funding for 25 young researchers. The European Union recognized and responded to the critical need for reinforcing research in ion therapy and the training of professionals in the rapidly emerging field of hadron therapy. The project was multidisciplinary, ambitious, and innovative; it had all the key partners, including the 2 European commercial leaders in the field. All this certainly did not come amiss and helped us to garner the funding for one of the most successful MC training projects where the globally recruited researchers trained are now the leaders in the field and still members of a very much alive collaborative network. B and C, Today, more than 10 years later, Particle Training Network for European Radiation therapy researchers continue to move forward and amaze us with their success in the hadron therapy community and their collaboration and leadership.

Twenty-nine young researchers throughout Europe, coming from a variety of backgrounds and countries, were trained.^3^, ^4^ Today, the PARTNER network is still very much alive. The then young researchers have all started to contribute with their expertise to various hadron therapy centers across the planet and have become the present experts who, in turn, are training other researchers to enter the field (Fig 4A and B).

PARTNER was over in 2012, but its legacy remains within ENLIGHT. Indeed, in 2015, at the annual meeting in Krakow, the members of the Network decided that training would become a regular element of each annual meeting. The participation to the ENLIGHT training courses organised in connection with the annual ENLIGHT meetings is delivered free of charge by ENLIGHT experts. So far, we can proudly say that we have trained hundreds of experts, and the number keeps growing each year.

## The 4 Projects

ULICE was a European Commission funded infrastructure project under the ENLIGHT umbrella and coordinated by Roberto Orecchia from CNAO. Launched in 2009, ULICE aimed at making hadron therapy more affordable and effective as well as fostering collaboration among existing and future centers.

Uncertainties in the actual range of the particle beam inside the patient and the patient set-up or dose calculation can reduce the accuracy during treatment. The problem is known as “quality assurance,” and can be mitigated by using advanced medical imaging techniques. To address this crucial challenge, in 2010, the European Commission funded a 4-year project called ENVISION. The project involved 15 research centers and one industrial partner.

Linked to ENVISION we launched ENTERVISION, a Marie-Curie Initial Training Network aimed at educating young scientists from the various fields—physics, medicine, electronics, informatics, radiobiology, and engineering—that are crucial to hadron therapy. Bringing together ten academic institutes and research centers of excellence and one industrial partner, ENTERVISION trained 15 young researchers. In 2013, the project was chosen as “a success story illustrating the good use of European funds for research” and “as a flagship project for Marie Curie Actions for the promotion of the H2020 programme, as a so-called ‘gold project.’”^7^

### Is the Model Reproducible?

ENLIGHT undoubtedly has contributed to foster the diffusion of hadron therapy in the global community, including in the United States. Thanks to ENLIGHT, Europe also has once again experienced first-hand the power of collaboration in creating a multidisciplinary field with the goal of empowering young generations through education. Other countries have witnessed the huge changes that the 3 pillars of ENLIGHT—collaboration, multidisciplinarity, and training—have produced in our continent.

## Can This be Reproduced Elsewhere?

The effects of ENLIGHT can be reproduced elsewhere, provided that the “I want to be the first” attitude does not prevail on the need for collaborating. In ENLIGHT, we see that other countries outside Europe like our approach and would like to reproduce it. We see it regularly at our meetings when experts from such countries look at the progress we make and the paths we find to support the field. However, we observe that in some cases there is too much competition, and the danger of that is that nothing will ever have been built, as that would require too many centers (and therefore too much money). Bringing people together, listening to their needs, and making them talk and look for common objectives has been the role of ENLIGHT in Europe and we are very proud of what we achieved.

One of the most enlightening initiatives that the network supported was the organization of conferences devoted to blending scientific backgrounds and expertise with the aim of creating a new culture of collaboration and sharing. The first of such conferences was Physics for Health in Europe (PHE), held in 2010 at CERN, the temple of fundamental physics. The first gatherings and conferences supported by ENLIGHT focused on the intrinsic physics processes that make hadron therapy a promising way of treating tumors which cannot be treated with conventional radiation therapy as they are either radio-resistant or located very close to critical organs. Topics related to radiobiology, accelerators, radioisotope production, detectors, and use of information technology were also discussed.

**Figure 5:**
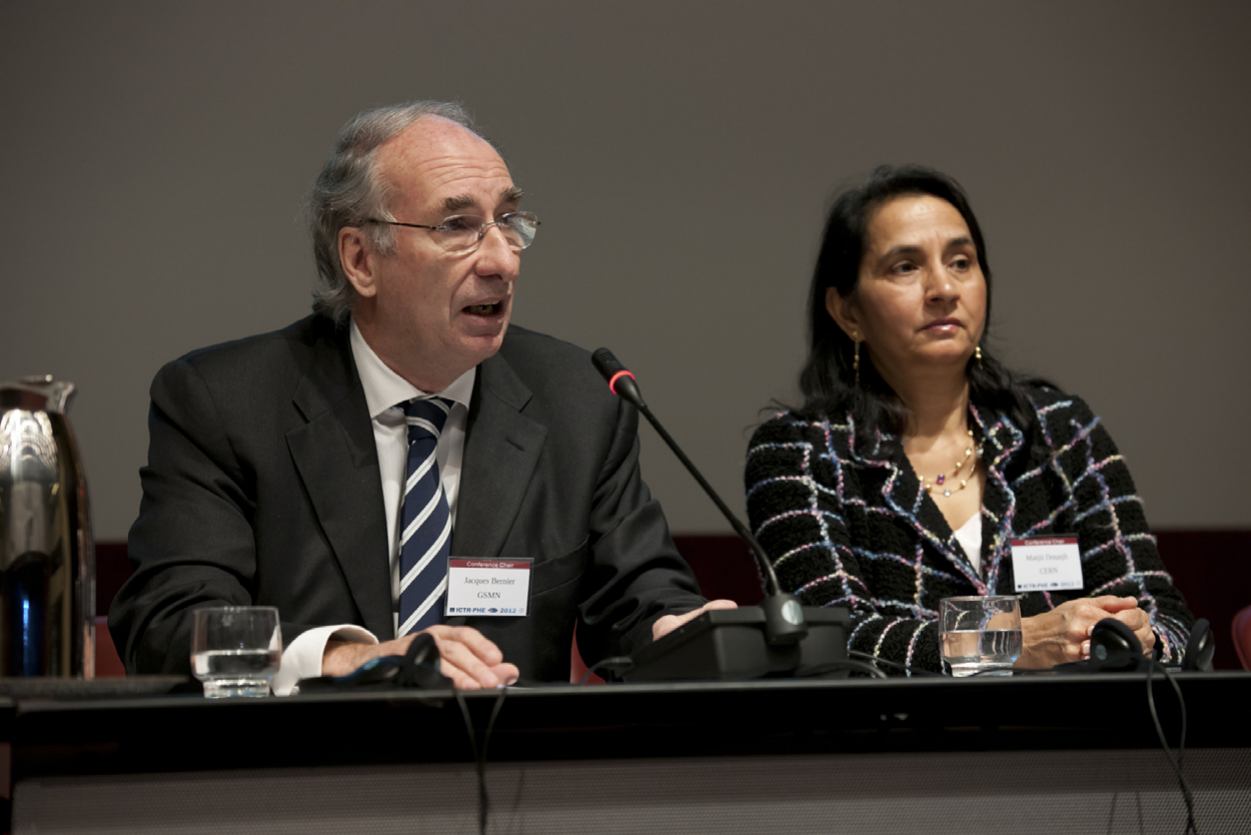
Manjit Dosanjh (right) with Jacques Bernier at the opening of the first International Conference on Translational Research in Radio-Oncology (ICTR)–Physics for Health in Europe (PHE) meeting that was built on and inspired from the European Network for Light Ion Hadron Therapy. The conference brought together the International Conference on Translational Research in Radio-Oncology and Physics for Health in Europe. ICTR-PHE became a winning alliance aiming to unite physics, biology, and medicine for better health care, and review the most recent advances in translational research, where developments in basic research are “translated” into means for improving health.^6^ As one of the speakers said, quoting the novelist William Gibson: “illiam Gibson “The future is already here – it's just not evenly distributed. *The Economist, December 4, 2003*”.^16^ This is the next challenge for the community of scientists who attended ICTR-PHE: how to quickly translate from “laboratory to bed.”

The seeds were there to rapidly extend and set more ambitious goals for the network. In a couple of years, the PHE conference had already united with the purely medical International Conference on Translational Research in Radio-Oncology (ICTR) to become the interdisciplinary ICTR-PHE conference, held every second year since 2012.^6^ With its 10-year long history and its already very impactful network of participants, ICTR injected new power into the newly created melting pot (Fig 5).

Just like ENLIGHT itself, ICTR-PHE was established as a multidisciplinary conference since its beginning, and it attracted experts in particle physics, hadron therapy, radiation therapy but also nuclear medicine, immunotherapy, etc. PHE alone could only deal with topics such as detectors and techniques to detect the effects of specific particles in the body.^8^ On the other hand, translational research was already in ICTR but only within the limits of x-rays. The 2 together created a new blend.

Over the years, the physicists learned to orient their efforts to best meet doctors’ needs, and the medical environment learned methodologies and approaches that came from the fundamental science labs. Sharing and discussing the results of the most recent research and addressing the challenges and possible developments to indicate the subjects with the highest priority for further studies in diagnosis and therapy on the European scale became just ordinary business for members of the community. A new way of thinking, working and collaborating was born and is still alive, despite the fact that the last ICTR-PHE conference was held in 2016.^9^

Today, we are going through very challenging times in public medicine, but we can also say that organizing conferences, even if only online, has become easier than it used to be.

## A Future for ENLIGHT and Hadron Therapy

In 2015, at the annual meeting in Krakow, the ENLIGHT members discussed the challenges and way forward for the network. There was clear agreement on the need of continuing such a broad umbrella organization. Education and training were defined as key activities for the future of ENLIGHT. An extended leadership support structure through a multidisciplinary advisory board was introduced to support the successful development of ENLIGHT.

More than 20 years after the start of ENLIGHT, we clearly observe that the focus for hadron therapy has naturally shifted from increasing the number of clinical centers to fostering the development of technologies that would ensure a safer and more cost-effective treatment.

**Figure 6:**
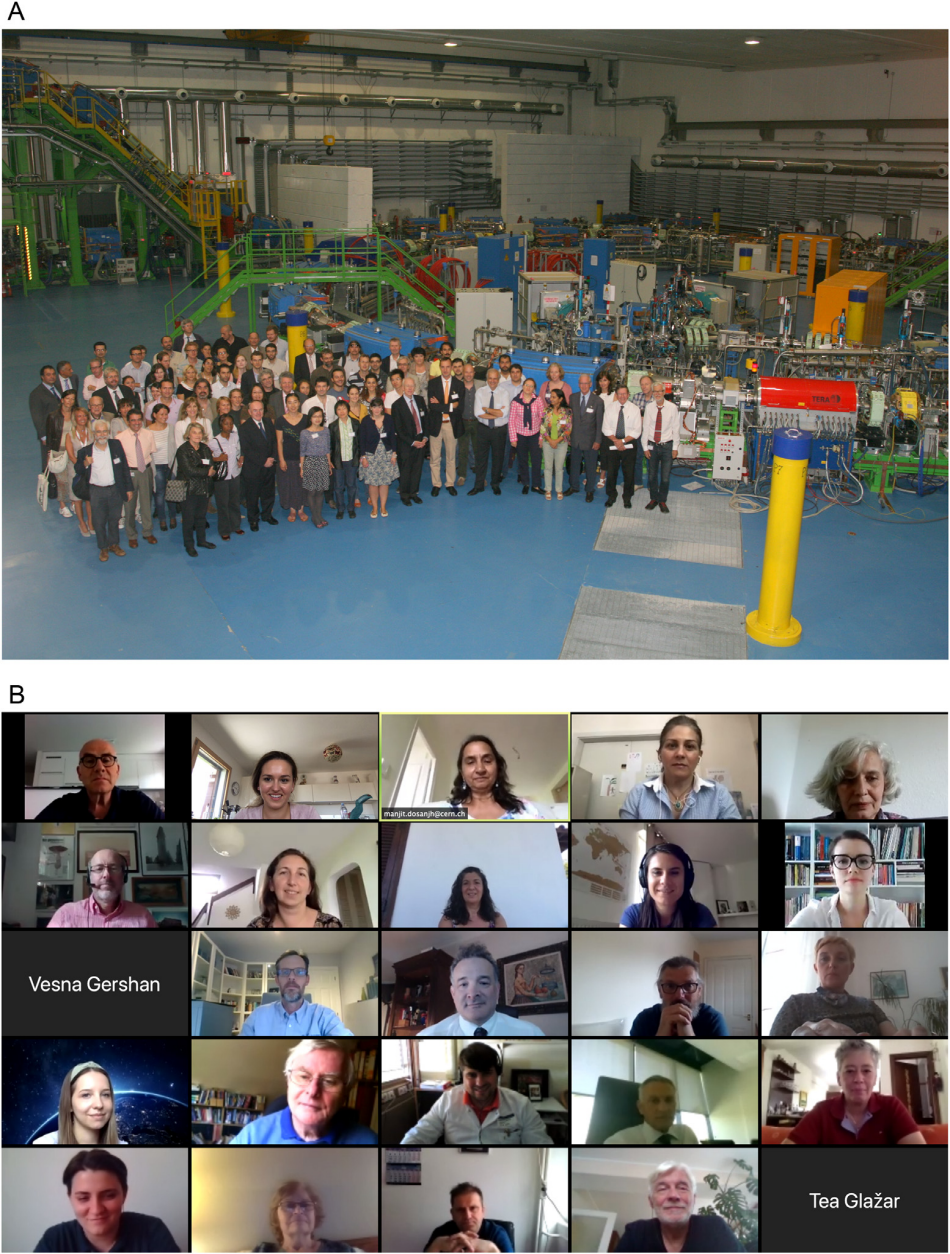
A and B, During these challenging COVID times, the European Network for Light Ion Hadron Therapy has not stopped reinventing itself in providing continuous support to the medical and physics communities. Toward this end an online meeting for the South-East European project, South-East European International Institute for Sustainable Technologies (www.seeiist.eu) was organized in the summer of 2020 to encourage the establishment of a community in South-East Europe who will share, develop, and lead to the successful implementation of hadron therapy in the region. The European Network for Light Ion Hadron Therapy leaders as ever rose to the challenge and readily provided their expertise and knowledge by giving the state-of-the-art talks to the audiences. The speakers included Sanja Damjanovic, Juergen Debus, Ugo Amaldi, Piero Fossati, Sandro Rossi, Eleanor Blakely, Robert Miller, and Maurizio Vretenar.

At present, ENLIGHT is being instrumental in supporting the South East European International Institute for Sustainable Technologies (SEEIIST), new international research infrastructure to be based in South-East Europe. Once again, CERN is acting as the catalyst and a politically neutral ground, bringing them all together. SEEIIST would be the first of its kind—Pan-European research infrastructure—with 50% of the beamtime dedicated to patient treatment and 50% to multidisciplinary research.^10^ Thanks to SEEIIST, cancer patients in the region will have access to state-of-the-art particle cancer therapy while scientists will be able to perform cutting-edge research and technology for the benefit of the whole region.^11^ ENLIGHT is supporting the project by fostering educational training and also sharing innovation. In 2020 our network organized a virtual meeting dedicated to SEEIIST. Our goal is to share knowledge and build a sustainable multidisciplinary and collaborative network of clinicians, medical physicists, physicists, computer experts, and engineering profiles to optimize the progress in cancer research and therapy to benefit all Europe. The project will help in the building of mutual trust in the SEE region in the spirit of “Science for Peace” as it will foster collaboration among all relevant stakeholders such as hospitals, universities, policymakers and industry, enabling rapid translation of research to the optimal treatment of cancer patients (Fig 6A and B).

Along the same lines, we are supporting the initiative of linear accelerators for other developing countries, including in Africa.^12^, ^13^, ^14^ The project,^15^ launched again at CERN, is called Smart Technologies to Extend Lives with Linear Accelerators and it aims at improving access to cancer treatment in those regions where there is either almost no care at all or where there is limited availability of Radiation therapy, which is often delivered using cobalt-60, a radioactive source that carries with it the potential problems of illicit use for terrorist threats as well as environmental and economic challenges related to source end-of-life disposal.

A novel, robust, modular linear accelerator that requires fewer staff and less maintenance while delivering state-of-the-art treatment is at the heart of the Smart Technologies to Extend Lives with Linear Accelerators projects, which will also make heavy use of artificial intelligence and deep learning to enhance the capability of the linear accelerator and to incorporate imaging and biological information into patient management, as well training and guiding the experts needed on the ground. For the whole project to work, the key element is a robust program of mentorship to help train, educate and sustain on-site expertise and treatment.

It is hard to say where the field will go in the next 5 to 10 years, but at the moment it appears clear that protons in particular are fairly mature as a technology, but not as a clinical practice. Indeed, in spite of a huge number of treatment centers across the globe, we have not yet treated enough patients with clinical protocols to let hadron therapy become an automatic part of cancer treatment. Hadron therapy will only become part of the radiation therapy practice when there will be enough facilities and enough patients for whom that is the right treatment based on sufficient clinical data and experience.

In parallel, one has to consider that the conventional radiation therapy with photons is also making huge progress. One excellent example is the new emerging Flash technology, which looks promising for the future also for its reduced cost with respect to hadron therapy. In addition, at the moment, the radiation therapy field is far from being totally exploited as we are currently focusing only in cancer treatment and not enough on other health conditions, such as tuberculosis, malaria, Alzheimer's, cardiovascular disease, and diabetes,

ENLIGHT and its powerful network of world specialists will continue to contribute to the growth of the particle therapy field. More ambitiously, I am confident that ENLIGHT will help in shaping that growth thanks to our “wisdom concept,” the win-win scenario in which the capacity of early and midcareer specialists is built using the life experiences and skills of the older generation of specialists.^14^ We firmly believe in our 3 pillars and their capacity to disrupt old habits for the benefit of society. It has not been easy so far, but we showed that it is possible, and we are eager to explore unchartered territories to prove that we can do even better in the future.
